# Smoking behaviour and health care costs coverage: a European cross-country comparison

**DOI:** 10.1007/s10754-017-9218-8

**Published:** 2017-05-30

**Authors:** Reza Rezayatmand, Wim Groot, Milena Pavlova

**Affiliations:** 1 Health Management and Economics Research Centre, Isfahan University of Medial Sciences, Isfahan, Iran; 2 Department of Health Services Research, Faculty of Health, Medicine and Life Science, CAPHRI, Maastricht University Medical Centre, Maastricht University, Maastricht, The Netherlands; 3 Top Institute for Evidence-Based Education Research (TIER), Maastricht University, Maastricht, The Netherlands

**Keywords:** Adverse selection, Smoking, Health insurance, Health care cost coverage, Instrumental variable, D820, I120, I130

## Abstract

The empirical evidence about the effect of smoking on health care cost coverage is not consistent with the expectations based on the notion of adverse selection. This evidence is mostly based on correlational studies which cannot isolate the adverse selection effect from the moral hazard effect. Exploiting data from the Survey of Health, Aging, and Retirement in Europe, this study uses an instrumental variable strategy to identify the causal effect of daily smoking on perceived health care cost coverage of those at age 50 or above in 12 European countries. Daily smoking is instrumented by a variable indicating whether or not there is any other daily smoker in the household. A self-assessment of health care cost coverage is used as the outcome measure. Among those who live with a partner (72% of the sample), the result is not statistically significant which means we find no effect of smoking on perceived health care cost coverage. However, among those who live without a partner, the results show that daily smokers have lower self-assessed perceived health care cost coverage. This finding replicates the same counter-intuitive relationship between smoking and health insurance presented in previous studies, but in a language of causality. In addition to this, we contribute to previous studies by a cross-country comparison which brings in different institutional arrangements, and by using the self-assessed perceived health care cost coverage which is broader than health insurance coverage.

## Introduction

Adverse selection, in the context of health insurance, refers to the phenomenon that individuals who anticipate higher health care expenditures are more likely to purchase health insurance or a more extensive coverage (Doiron et al. 2008). A test for the presence of adverse selection is whether people with a higher health risk (e.g. because they smoke) choose for more comprehensive health insurance coverage. However, a reverse effect is also possible which is termed as ex-ante moral hazard. This refers to a situation where a comprehensive health insurance scheme creates an incentive for engaging in unhealthy behaviours such as smoking. Thus, the observed correlation between health insurance and health risk can be a composite of both the adverse selection and the moral hazard effect.

Several authors have tested the adverse selection hypothesis by using unhealthy behaviour like smoking as an indicator for health risk. But their conclusions contradict the adverse selection explanation. For instance, studies by Hopkins and Kidd (1996), Barrett and Conlon (2003) and Doiron et al. (2008) show that in Australia smokers are less likely to buy private health insurance. Doiron et al. (2008) provide evidence that engaging in risky behaviour is positively correlated with being in worse health. They conclude that the decision to buy private health insurance is more affected by other factors such as risk aversion, income or socioeconomic status than adverse selection. In other words, high risk aversion jointly determines the decision to buy private health insurance and the decision not to engage in high risk health-related behaviour like smoking. At the same time, it is argued that smokers might behave in a myopic manner, implying that they tend to ignore the future consequence of their current consumption (Becker et al. 1994). As a result, these individuals do not consider costs incurred in the future and therefore do not buy a (more extensive coverage in) health insurance. A recent study by Lambert et al. (2011) among the elderly in Europe using SHARE dataset exploit a different health risk indicator to track any evidence of adverse selection between health risk and health care cost coverage (Lambert et al. 2011). They use the number of long term spells of ill health in the past as a health risk indicator. Their study shows no evidence of adverse selection in health insurance among European elderly people.

All above mentioned studies on adverse selection are correlational studies which fail to identify a casual effect. Moreover, the methodological techniques that are used in those studies could not appropriately address the endogeneity of smoking behaviour. In this study, however, we use an instrumental variable strategy (IV) to deal with this simultaneous causality bias and to identify a causal effect. We postulate that smoking is perceived as a health risk by individuals, and potentially affect their health care cost coverage along with other factors like age, gender, education, economic status, and health status. To our knowledge, this is the first study which uses IV to examine the causal effect of smoking on perceived health care cost coverage (i.e. the adverse selection perspective).

Also, most empirical evidence about the relationship between unhealthy behaviour (e.g. smoking) and health insurance is limited to one country (Barrett and Conlon 2003; Doiron et al. 2008; Hopkins and Kidd 1996). Hence, their results may not be generalized to other countries. However, this study uses data from the Survey of Health, Aging, and Retirement in Europe (SHARE) which allows for a comparison across European countries included in SHARE. This way, we are able to explore the extent to which the context of each country is related to the adverse selection effect. The work of Lambert et al. (2011), which has also exploited SHARE data, uses a different health risk indicator (namely the number of long term spells of ill health in the past). Thus, our study can explore how a different health risk indicator (namely daily smoking) can make a difference.

Health care cost coverage is a broader concept than health insurance coverage. The latter is just one way of offering health care cost coverage. Health care coverage can have three dimensions: breadth, the proportion of the population covered by the health care system; scope, the generosity of the benefit package (the services/goods covered); and depth, the level of coverage (the proportion of cost covered) (Teutsch and Rechel 2012). The institutional arrangements regarding the above dimensions vary between countries. The following background section provides detailed information on specific institutional arrangements regarding health care cost coverage in European countries. It shows that out of specific institutional arrangement in each country, in most cases, full coverage is likely to be offered at the individuals’ discretion. This means that individuals can decide themselves whether or not to have full health care cost coverage. This is the main assumption we are making and analysing here.

## Background

Health coverage and financial arrangements are fundamental characteristics of a health system. The WHO has called for universal coverage to assure access to health care for everyone in need (WHO 2010). We use the results of an OECD survey on health system characteristics (Paris et al. 2010) to outline the three dimensions of health care cost coverage among the countries that we study (Table 1).

**Table 1:** Characteristics of basic primary health coverage. *Source* Composed by the authors based on information provided in Paris et al. (2010)

Country	Types of basic primary health coverage (% of population)			The basic health coverage is provided by/consumer’s choice of insurer if multiple insurer are involved	Private health insurance (% of population)			Coverage(%) of health services by basic primary health coverage				Basic health care cost coverage score
	Automatic	Compulsory	Voluntary		Duplicate	Complementary	Supplementary	Acute inpatient care	Outpatient primary physician contact	Outpatient specialist contact	Pharmaceuticals	
Austria	0	98.7	0	Multiple insurers/no	0	33.3	33.3	76–99	100	100	76–99	14
Belgium	0	99	0	Common health insurance scheme	0	77.4	77.4	76–99	76–99	76–99	76–99	12
Czech Republic	0	100	0	Multiple insurers/yes	0	0	0	76–99	76–99	76–99	51–75	11
Denmark	100	0	0	Local health services	0	15.5	15.5	100	100	100	51–75	14
France	2.5	97.5	0	Multiple insurers/no	0	92	92	76–99	51–57	51–57	51–75	9
Germany	0.5	83.3	15.2	Multiple insurers/yes	0	17.5	17.5	100	76–99	76–99	76–99	13
Greece	0	100	0	Multiple insurers/no	8	0	0	76–99	76–99	76–99	76–99	12
Italy	100	0	0	National health services	15.6	0	0	100	100	76–99	100	15
Netherlands	0	100	0	Multiple insurers/yes	0	0	92	100	100	100	100	16
Poland	0	99	0	Common health insurance scheme	2.6	0	0	100	100	100	51–75	14
Spain	100	0	0	Local health services	10.3	0	0	100	100	100	76–99	15
Switzerland	0	100	0	Multiple insurers/yes	0	0	29.5	100	76-99	76–99	76–99	13

As depicted in Table 1, nearly all residents in the countries included in our study are covered by a basic scheme. This coverage is provided either automatically (Denmark, Italy, Spain) or compulsorily (in other countries). Germany is an exception in the way that high-income people can voluntarily choose a private health insurance instead of social insurance for their basic coverage. Automatic coverage is provided by national (Italy) or local health services (Denmark, Spain) which are financed from taxes. Social health insurance schemes which are financed through income-related social contributions usually provide compulsory coverage but in a slightly different ways across the countries. For instance, in Belgium and Poland there is only one health insurance scheme, while in the other countries there are multiple insurers. In some of these countries, people are allowed to choose their insurer (Czech Republic, Germany, the Netherlands, Switzerland) while in others, there is no choice of insurer, and nevertheless there are multiple insurers (Austria, France, Greece). In the Netherlands and Switzerland, health insurance is compulsory for all, but individuals pay community-rated premiums to private health insurance funds.

Most countries guarantee a high level of coverage particularly for acute inpatient and outpatient (primary physician and specialist contact). France is singular in that it covers only 60% of outpatient physician services. Pharmaceuticals are typically covered at lower levels in most countries except in the Netherlands and Italy where they are fully covered. In order to compare coverage between countries, using the OECD data, we create an indicator based on the level of coverage for the above mentioned services. We assign codes 4 to 1 to the level of coverage 100, 76–99, 51–75 and 50% or less respectively (as defined in the OECD survey). Thus, a country gets a number of 16 if it fully covers the four health care services mentioned in Table 1. These results are presented in the last column of Table 1. For instance, according to this indicator, we can see that the highest coverage is provided in the Netherlands followed by Spain. In contrast, the lowest coverage is being provided in France. However, it is worth noting that this indicator provides information only about the basic health care cost coverage.

Almost all countries in our study also have institutional arrangements for private health insurance as a secondary source of coverage. Private health insurance can be complementary (covering the user fees), supplementary (increasing the benefit package) or duplicative (providing a quicker access to health care services). As depicted in Table 1, complementary private health insurance plays a prominent role in France, filling the gap in coverage for outpatient primary physician and specialist contacts. Supplementary private health insurance is held by 92% of people in the Netherlands. The Czech Republic and Greece report a zero proportion of their population having either complementary or supplementary private health insurance, while 8% of the Greek citizens obtain duplicate private health insurance.

## Methods

### Data and study sample

The Survey on Health and Retirement in Europe (SHARE) is a multidisciplinary and cross-national panel dataset with micro-level data on health, socioeconomic status as well as social and family networks. It is based on nationally representative samples of more than 85,000 individuals aged 50 or over in Europe (SHARE 2012). The SHARE baseline study was conducted in 11 countries in 2004, followed by a second wave in 2006 in 14 countries. The third wave of the survey, SHARELIFE, which was conducted in 2008–2009, only collected detailed retrospective data on life histories in 13 countries. A fourth wave (comparable to wave 1 and 2) has been performed in 2011 in 19 countries. In this study, we use data from the second wave (release 2.5.0) of SHARE. We could not include the first and the fourth waves of the SHARE data because the questions regarding health insurance coverage that we use in our analysis either have not been asked (first wave) or have been asked in a different way (by self-administered questionnaire instead of asking by an interviewer in fourth wave). As noted earlier, the third wave does not provide any information relevant to our analysis.

In total, 14 countries are included in the SHARE wave 2, namely; Austria, Germany, Sweden, Netherlands, Spain, Italy, France, Denmark, Greece, Switzerland, Belgium, Czech Republic, Poland, and Ireland. We had to exclude Ireland and Sweden from our study because the imputed variables for annual household net income generated by SHARE were not available for Ireland and the number of missing value for perceived health care cost coverage was considerable in Sweden. Thus, overall, our sample consists of 30,536 individuals from 12 countries.

### Perceived health care cost coverage variables

In wave 2, the SHARE respondents are asked about their health care cost coverage. In order to measure the individual’s health care cost coverage, the interviewer is instructed to name different types of health care services ranging from visiting a general practitioner to nursing care at home in case of chronic diseases or disability. Then, the interviewer asks each individual to identify who finally pays for these services by choosing one option from the following four options: 1 = entirely paid by respondent, 2 = mostly paid by respondent, 3 = mostly paid, or reimbursed by social insurance and/or respondent’s health insurance, and 4 = entirely paid, or reimbursed by social insurance and/or respondent’s health insurance. For each respondent, we sum up the codes of the response options indicated by the respondent for each service (medical visit to general practitioner, medical visit to specialist when prescribed by general practitioner, prescribed drug, and hospitalization in public hospital) to create a variable indicating the extent of self-assessed perceived health care cost coverage. This variable ranges from 4 for those who entirely have to pay for the above mentioned services themselves to 16 for those who will get fully reimbursed by their social insurance or/and their own health insurance. It should be noted that this variable provides a self-assessment of the extent of the health care cost coverage which might not correspond to the actual coverage. However, in order to affect individuals’ behaviours, the coverage assessed by the individuals is likely to matter more than the actual coverage. Therefore, it is suitable for our analysis. As mentioned earlier, in wave four, the same questions as in the second wave are asked about the health care cost coverage but by means of a self-administered questionnaire. Thus, we could not include this wave both because of the large number of missing values, and because of the inconsistency in data collection compared with the second wave.

### Analytical methods

The IV estimation in our study uses daily smoking as the endogenous variable. The IV approach involves finding at least one exogenous observable variable (called the instrument) that is highly correlated with the endogenous variable but is not correlated with the error term of the outcome equation (perceived health care cost coverage). In our study, self-assessed perceived health care costs coverage is the outcome variable. The IV estimation consists of a two-step process. First, the endogenous variable is run against all covariates including the instrument. Then, the predicted value of the endogenous variable-instead of the actual value- is used in the second stage. Because our variable of interest for daily smoking is binary (yes/no), using a linear model in the first stage is not appropriate. Thus, we fit a treatment-effect model using full maximum likelihood which estimates two regressions simultaneously. The first equation is estimated using probit regression to predict the probability of daily smoking. The second is a linear regression for perceived health care cost coverage. The two error terms are assumed to be jointly normally distributed. More details about the specification of a treatment-effect model can be found in Cameron and Trivedi (2009) and Khandker et al. (2010).

Thus, borrowing the notation of Cameron and Trivedi (2009), the treatment-effect model can be written as:

$$\begin{aligned} y_{1i}= & {} \beta _1 {\hbox {X}^{\prime }}_{1i} +\beta _2 y_{2i} +u_i \qquad \qquad \left( {Second\,stage\,regression:Linear\,regression}\right) \\ {y^{*}}_{2i}= & {} \pi _{1j} {\hbox {X}^{\prime }}_{1i} +\pi _{2j} {\hbox {X}^{\prime }}_{2i} +v_i \qquad \qquad \left( {First\,stage\,regression: Probit} \right) \\ y_{2i}= & {} \left\{ {{\begin{array}{l} {1\,if\,{y^{*}}_{2i} >0} \\ {0\,otherwise} \\ \end{array} }} \right\} \end{aligned}$$

where $y_{1}$ is the perceived health care cost coverage, $y_{2}$ is daily smoking (treatment indicator), $X_{2}$ are our instrumental variables (i.e. whether the spouse or partner is a daily smoker for those who are living with a partner, and whether there is any other daily smoker in the household (aside from the respondent him/herself) for those who do not live with a partner), $X_{1}$ are exogenous variables in the models (i.e. age, years of education, annual household income (LN), self-perceived health, the score on the global limitation index (GALI), If there is a child aged ${\le }18$ in the household), $\beta$ and $\pi$ are regression coefficients.

### Identification strategy

According to the health capital model, smoking can be considered as a negative investment in health capital. However, the model does not account for the fact that this decision is not made in isolation. Peer effects in health behaviour have been investigated in a rapidly growing empirical literature (Cawley and Ruhm 2012). In particular, there is a large body of evidence on concordant health behaviours among couples (Graham and Braun 1999; Meyler et al. 2007). One study shows that a partner who smokes influences the other’s smoking behaviour. This study has also found more support for husband’s influence than the wife’s influence (Homish and Leonard 2005). Another study has reported that smoking cessation by a spouse decreases the other one’s chance of smoking by 67%, while the smoking cessation by a sibling decreases the chances only by 25% (Christakis and Fowler 2008). Thus, not only social ties but also the nature of social ties may play a role in smoking behaviour. Given these facts, two instrumental dummy variables are created: whether the spouse or partner is a daily smoker for those who are living with a partner, and whether there is any other daily smoker in the household (aside from the respondent him/herself) for those who do not live with a partner. By the term “with a partner” we refer to both legally married couples and those who live together. It is expected that these variables are correlated with smoking behaviour but they are not correlated with perceived health care cost coverage (we test whether this assumption is correct). The following part explains different methods used to test the quality of instruments.

### Instrument validity and relevance

An instrumental variable must be relevant, meaning that it should have a strong association with the endogenous variable. A weak instrument is only marginally relevant and explains little of the variation in the endogenous variable. This can be tested by obtaining the F statistics of the instrument in the first stage regression. The widely used rule of thumb considers an F statistics of more than 10 as indicating a strong enough instrument (Cameron and Trivedi 2009). As in our analysis the first stage regression is a probit regression, we present the corresponding chi square test instead of the F test in the first column of Table 2. These test statistics are considerably larger than 10 in all models.

**Table 2:** Instrument quality tests. *Source* Authors’ analysis of SHARE wave 2

Model	Females			Males		
	1	2	3	1	2	3
Living with partner	401.74	0.03	$-$0.01	397.98	1.88	$-$.003
Living without partner	15.01	0.50	0.025	11.24	0.01	0.018

(1) is the chi 2 test for the exclusion of the instrument in the first stage equation. (2) is the F test for the exclusion of the instrument in the second-stage equation. (3) is the spearman correlation coefficient between health insurance coverage and the instruments

An instrument must be valid too, meaning that it should not be correlated with the error term in the equation of interest. This condition is not possible to test in a just identified model, such as our model, and should be judged based on information that is extraneous to the data (Morgan and Winship 2007; Rose and Stone 2011). However, as done by Trostel et al. (2002), we conduct a F test of the effect of the instrument on the perceived health care cost coverage residual to ensure that the instruments are not directly correlated with the perceived health care cost coverage once the other exogenous variables are included. These are reported in the second column of Table 2. As can be seen, these tests do not appear statistically significant in all models which implies no correlation between the residuals and the instrument. In order to get more insight, we also report the Spearman correlation coefficient between perceived health care cost coverage and our instruments. As depicted in the third column of Table 2, the correlation coefficients are either zero or very close to zero in all models which indicates no or very weak correlation between the perceived health care cost coverage and our instrument.

**Table 3:** Descriptive statistics. *Source* Authors’ analysis of data from SHARE wave 2

	Austria mean (SD) N	Belgium mean (SD) N	Czech Republic mean (SD) N	Denmark mean (SD) N	France mean (SD) N	Germany mean (SD) N	Greece mean (SD) N	Italy mean (SD) N	Netherlands mean (SD) N	Poland mean (SD) N	Spain mean (SD) N	Switzerland mean (SD) N	Total mean (SD) N
Daily smoking	0.15	0.17	0.21	0.27	0.14	0.17	0.29	0.17	0.22	0.26	0.15	0.19	0.20
Yes $=$ 1	(0.36)	(0.38)	(0.41)	(0.44)	(0.35)	(0.37)	(0.45)	(0.38)	(0.42)	(0.44)	(0.36)	(0.39)	(0.40)
No $=$ 0	1332	3139	2801	2558	2905	2541	3236	2975	2619	2448	2216	1446	30216
Self-assessed health care coverage	13.71	11.96	14.46	14.37	14.09	13.37	13.73	13.82	15.68	13.56	14.96	12.78	13.88
	(1.99)	(1.11)	(1.21)	(1.08)	(2.02)	(1.87)	(2.11)	(2.02)	(0.83)	(1.45)	(1.7)	(1.95)	(1.90)
	1322	2982	2656	2472	2665	2451	3194	2923	2580	2319	2190	1421	29175
Age	66.82	65.02	63.84	64.17	64.36	65.04	64.43	65.63	64.18	64.16	66.76	64.74	64.82
	(9.62)	(10.66)	(10.01)	(10.7)	(11.16)	(9.65)	(10.89)	(9.69)	(9.93)	(10.21)	(10.99)	(10.68)	(10.42)
	1341	3169	2830	2616	2968	2568	3242	2983	2661	2467	2228	1462	30536
Gender Male $=$ 1 Female $=$ 0	0.41	0.45	0.42	0.45	0.43	0.46	0.43	0.45	0.46	0.44	0.45	0.44	0.44
	(0.49)	(0.50)	(0.49)	(0.50)	(0.50)	(0.50)	(0.50)	(0.50)	(0.50)	(0.50)	(0.50)	(0.50)	(0.50)
	1341	3169	2830	2616	2968	2568	3243	2983	2661	2467	2228	1462	30536
Living with spouse or partner yes $=$ 1 no $=$ 0	0.59	0.71	0.64	0.68	0.66	0.76	0.73	0.81	0.77	0.74	0.76	0.67	0.72
	(0.49)	(0.45)	(0.48)	(0.47)	(0.47)	(0.43)	(0.44)	(0.39)	(0.42)	(0.44)	(0.43)	(0.47)	(0.45)
	1341	3169	2830	2616	2968	2568	3243	2983	2661	2467	2228	1462	30536
Child ${\le }18$ years in household yes $=$ 1 no $=$ 0	0.05	0.05	0.06	0.08	0.10	0.05	0.06	0.07	0.07	0.20	0.07	0.07	0.08
	(0.21)	(0.22)	(0.24)	(0.27)	(0.3)	(0.21)	(0.24)	(0.26)	(0.26)	(0.4)	(0.25)	(0.26)	(0.26)
	1341	3169	2830	2616	2968	2568	3243	2983	2661	2467	2228	1462	30536
Hhd annual net income €	26894	31415	15846	31638	37206	34406	22314	25052	41650	11577	22788	42371	28203
	(9838)	(36032)	(14058)	(21520)	(36546)	(37733)	(23031)	(23807)	(46965)	(11428)	(29447)	(38805)	(31480)
	1341	3169	2830	2616	2967	2568	3243	2983	2661	2467	2228	1462	30533
Years of education	7.5	11.7	11.67	7.95	11.17	12.48	8.49	7.86	11.05	9.2	6.97	11.11	9.85
	(4.97)	(3.68)	(3.03)	(5.68)	(4.14)	(3.25)	(4.29)	(4.38)	(3.73)	(3.32)	(5.07)	(4.87)	(4.57)
	1330	3165	2823	2608	2931	2559	3188	2975	2646	2461	2205	1451	30342
Self-perceived health from poor $=$1 to excellent $=$ 5	2.97	3.01	2.66	3.41	2.81	2.8	3.13	2.7	3.06	2.13	2.55	3.4	2.9
	(1.03)	(1.02)	(1)	(1.17)	(1.03)	(1.02)	(1)	(1.08)	(1.04)	(0.99)	(0.97)	(1.03)	(1.1)
	1341	3169	2830	2616	2967	2568	3243	2983	2661	2467	2228	1462	30535
Global limitation index(GALI) limited $=$1 non-limited $=$ 0	0.13	0.16	0.19	0.12	0.16	0.17	0.07	0.13	0.19	0.28	0.07	0.09	0.15
	(0.34)	(0.36)	(0.39)	(0.33)	(0.36)	(0.37)	(0.25)	(0.34)	(0.39)	(0.45)	(0.26)	(0.29)	(0.35)
	1341	3169	2830	2616	2967	2568	3243	2983	2661	2467	2228	1462	30535

### Exogenous variables

We also include other exogenous variables in the models. These include age, education, self-perceived health, and a dummy variable indicating the Global Activity Limitation Index (GALI). Table 3 shows all explanatory variables and their coding. For income, we use the variable generated in the SHARE dataset for household annual net income. The SHARE dataset provides five imputed values for each missing household annual net income (SHARE 2011). We use the median of these five values. The income variable is then adjusted based on purchasing power parity in 2005 in Germany. The logarithmic function of the adjusted value is used as our explanatory variable for income. For education, we use a continuous variable indicating years of education. We include two variables to proxy health status. The first is a self-rated question indicating self-perceived health ranging from poor to excellent. The second is a dummy variable indicating each individual’s limitation on activity because of a health problem [the so-called GALI (Jagger et al. 2010)]. Country dummies (with reference to the Netherlands) are also included to account for contextual factors of each country. However, in order to check the robustness of the results, a per-country analysis is also performed for each model.

## Results

### Descriptive statistics

As depicted in Table 3, on average, 20% of the respondents in our sample are a daily smoker. Greece and France have the highest and the lowest prevalence of daily smokers (29 and 14%), respectively. With regard to perceived health care cost coverage, the Netherlands followed by Spain provide the most generous coverage (similar to our estimations based on OECD data, see Table 1). Regarding demographic factors, on average, our sample is at the age of 65, 44% is men and 72% is living with a spouse or a partner and 19% is living alone. As expected, the socioeconomic factors vary among the countries included. Annual household net income, adjusted for purchasing power parity, in Poland (11,577 Euro) is almost a quarter of the annual household net income in Switzerland (42,371 Euro). There is also a large difference between countries with regard to the level of education. While respondents in Spain, on average, report 7 years of education, those who live in Germany, report nearly 12.5 years of education. With regard to self-perceived health status, Swiss and Danish people have the highest perceived health status, while the Polish self-rate their health status, on average, as fair on a scale from poor to excellent.

### Perceived health care cost coverage and daily smoking

As noted in the method section, based on marital status, we use two variables to instrument smoking, that is; whether the partner smokes for those who live with a partner (our first model) and whether another household member smokes for those who live without a partner (our second model). For each model, a gender stratified analysis is performed. All results should be interpreted taking into account the fact that our sample primarily consists of individuals aged 50 or over.

**Table 4:** IV and OLS estimates. *Source* Authors’ analysis of SHARE wave 2

Model: treatment effect	Second stage regression				First stage regression			
	Living with partner		Living without partner		Living with partner		Living without partner	
	Female	Male	Female	Male	Female	Male	Female	Male
Dependent variable	Perceived health care cost coverage				Daily smoking			
Instrumental variables								
If partner smoker					0.691***	0.733***		
					(0.035)	(0.038)		
If Other household member smoker							0.322**	0.269**
							(0.103)	(0.103)
Instrumented variable								
Daily smoking	0.128	$-$0.335	$-$2.150***	$-$2.337***				
	(0.131)	(0.254)	(0.111)	(0.150)				
Model: OLS								
Daily smoking	$-$0.046	$-$0.048	$-$0.053	$-$0.013				
	(0.044)	(0.039)	(0.062)	(0.082)				
*N*	10511	10379	5539	2340	10511	10379	5539	2340

Other exogenous variables in the model include: Age, years of education, annual household income (LN), self-perceived health, global limitation index (GALI), If child aged ${\le }18$ in the household, and country dummies

Standard error in parenthesis,* *p* value${\le }0.05$, ** *p* value${\le }0.01$, *** *p* value${\le }0.001$

As depicted in the left part of Table 4 (i.e. second stage regression), the effect of smoking on perceived health care cost coverage is not statistically significant for individuals who live with a partner. However, for those who live without a partner, smoking has a negative impact on perceived health care cost coverage, that is being a daily smoker decreases perceived health care cost coverage by 2.15 and 2.33 units for women and men, respectively. For a comparison, the results of regressing perceived health care cost coverage on daily smoking using OLS estimation show coefficients that are not statistically significant (see Table 4).

**Table 5:** IV estimates per country. *Source* Authors’ analysis of SHARE wave 2

	Female with partner		Male with partner		Female without partner		Male without partner	
Stage of regression	Second	First	Second	First	Second	First	Second	First
Dependent variable	Health cost coverage	Daily smoking	Health cost coverage	Daily smoking	Health cost coverage	Daily smoking	Health cost coverage	Daily smoking
Independent variable	smoke	If partner smoker	smoke	If partner smoker	smoke	If other household member smoker	smoke	If other household member smoker
Austria	1.141	1.003***	$-$0.599	0.941***	0.182	$-$0.272	1.874	$-$4.878
	(0.640)	(0.193)	(1.142)	(0.205)	(1.182)	(0.670)	(1.415)	(3029.64)
Germany	0.423	0.96***	$-$0.376	1.068***	0.272	1.282*	$-$1.996	1.466*
	(0.453)	(0.129)	(0.577)	(0.143)	(0.722)	(0.537)	(1.574)	(0.578)
Netherland	$-$0.989***	0.669***	0.19	0.939***	$-$0.494	0.436	0.441	0.206
	(0.092)	(0.101)	(0.145)	(0.117)	(0.290)	(0.477)	(0.436)	(0.394)
Spain	$-$0.712	0.406*	$-$2.694***	0.108	$-$2.35***	$-$0.198	$-$0.498	$-$0.5
	(0.51)	(0.161)	(0.190)	(0.148)	(0.284)	(0.452)	(0.768)	(0.676)
Italy	0.091	0.628***	$-$2.536***	0.55***	$-$3.422***	0.095	$-$3.403***	$-$0.423
	(0.664)	(0.112)	(0.319)	(0.112)	(0.417)	(0.400)	(0.495)	(0.369)
France	$-$0.282	0.55***	$-$3.818***	0.246**	0.311	0.293	1.129	0.081
	(0.629)	(0.153)	(0.322)	(0.123)	(1.183)	(0.376)	(1.063)	(0.422)
Denmark	$-$0.19	0.999***	$-$1.522***	0.614***	0.282	0.616*	0.648	0.725*
	(0.206)	(0.111)	(0.162)	(0.104)	(0.393)	(0.296)	(0.477)	(0.353)
Greece	0.189	0.365***	$-$0.689	0.352***	$-$1.216	1.476	$-$3.143***	5.62
	(1.17)	(0.090)	(0.85)	(0.094)	(1.091)	(0.798)	(0.673)	(0.000)
Switzerland	0.336	1.005***	$-$0.386	0.944***	$-$0.578	6.218	2.067*	1.057*
	(0.679)	(0.177)	(0.748)	(0.187)	(1.051)	(936.449)	(0.958)	(0.465)
Belgium	$-$0.404	0.647***	$-$0.078	0.71***	$-$1.483***	0.14	$-$1.214**	0.521
	(0.35)	(0.113)	(0.272)	(0.127)	(0.144)	(0.347)	(0.459)	(0.374)
Czech Republic	$-$1.686***	0.566***	$-$1.545***	0.68***	$-$0.07	0.594*	$-$1.594***	0.607**
	(0.197)	(0.105)	(0.178)	(0.122)	(0.309)	(0.277)	(0.359)	(0.227)
Poland	0.436	0.388**	$-$0.851	0.402*	1.104**	$-$0.537	$-$0.2	$-$1.066
	(0.425)	(0.120)	(0.480)	(0.124)	(0.523)	(0.817)	(1.396)	(0.823)

Other exogenous variables in the model include: Age, years of education, annual household income (LN), self-perceived health, global limitation index (GALI), If child aged ${\le }18$ in the household

Standard error in parenthesis,* *p* value ${\le }0.05$, ** *p* value${\le }0.01$, *** *p* value ${\le }0.001$

However, we observe some difference across the countries based on the results from our analysis per country (see Table 5) when compared with the above all-countries results (see Table 4). The first column of Table 5 shows for each group the IV estimates of the effect of daily smoking on perceived health care cost coverage per country. As noted earlier, the average effect of smoking on perceived health care cost coverage among women who live with a partner is not statistically significant in the all-countries model (Table 4). However, as reflected in Table 5, daily smoking has a negative effect on perceived health care cost coverage among women living with a partner in the Netherlands and Czech Republic. For men living with a partner, the findings show a considerable negative effect of smoking on perceived health care cost coverage in Spain, Italy, France, Denmark, and Czech Republic (Table 5), while at the same time the average effect of smoking in the all-countries model is not statistically significant (Table 4). Among those who live without a partner, our results show an overall negative effect of smoking on perceived health care cost coverage for both men and women (Table 4). However, referring to the country specific results, we see a positive effect for women in Poland and for men in Switzerland (Table 5).

With reference to country specific results (Table 5), having a smoker as a partner is associated with daily smoking among women in all countries, although the size of effect is not the same across different countries. The same also applies for men in all countries except Spain where the partner’s smoking behaviour seemingly has no effect on men’s smoking behaviour. With regard to the effect of having any other daily smoker in the household on the smoking behaviour of women without a partner, the results appear statistically significant in fewer countries (i.e. Germany, Denmark, and Czech Republic). It is also the case for men without a partner in Germany, Denmark, Switzerland, and Czech Republic who are more likely to be a daily smoker if there is another smoker in the household.

## Discussion

### Perceived health care cost coverage and smoking behaviour

In this study, we use an IV strategy to investigate the effect of daily smoking on perceived health care cost coverage of the elderly (aged 50+) in 12 European countries. Our findings show that smoking has a negative effect on self-assessed perceived health care cost coverage but only among those who live without a partner. For those who live with a partner (72% of the sample), our results are not statistically significant, which means there is no effect of smoking on perceived health care cost coverage. Overall, regarding the effect of smoking on perceived health care cost coverage; we either have no effect or an effect which is not compatible with adverse selection interpretation. Since among those (men and women) who live without a partner, smoking decreases self-assessed perceived health care cost coverage. It should be emphasized again that our variable of interest for perceived health care cost coverage is a self-assessment of the coverage (i.e. what individuals perceive as health coverage they would have when seeking health care services), and our sample primarily includes individuals aged 50 or over.

Most of the previous evidence about the effect of smoking on health insurance originated from Australia (Barrett and Conlon 2003; Doiron et al. 2008; Hopkins and Kidd 1996). They are mostly correlational studies about the effect of smoking on the demand for private health insurance to see if it is in accordance with adverse selection effect. However, they show that smokers are less likely to buy insurance, which is contrary to the theoretical expectations based on adverse selection. Those findings have been explained by authors through heterogeneity in risk aversion (Doiron et al. 2008). In other words, they explain that the decisions to buy private health insurance and to smoke are jointly determined by risk aversion. Alternatively, it may also be explained by myopic behaviour of smokers (Becker et al. 1994). Similar to previous studies, we also find a negative impact of smoking on perceived health care cost coverage but only among those living without a partner. We contribute to previous results, first, by studying this effect among different countries with different institutional arrangements for perceived health care cost coverage. Second, a broader sense of health care cost coverage rather than just health insurance coverage is taken into account. As previously noted, health care cost coverage has three dimensions (i.e. breadth, scope, and depth). In fact, all those dimensions are simultaneously reflected in the variable we use, since it is a self-assessment of what individuals perceive as health care cost coverage for all health care services, either covered by basic or voluntary private insurance or uninsured services. Our third contribution is to identify a casual effect by using an IV strategy, which has not been done before in studies focused on this topic.

### Adverse selection and European health care systems

Adverse selection occurs when individuals have a choice whether or not to buy health insurance. With choice, a positive relationship between (health) risk and insurance coverage can be expected (Cutler and Zeckhauser 2000). The same applies when we interpret the negative relationship between (health) risk (e.g. smoking) and insurance by heterogeneity in risk aversion between individuals. In this case, more risk-taking individuals (smokers) are also more likely to take more risk in their health insurance coverage and opt for less coverage. This argument holds even if we consider myopic behaviour for smokers. However, one might argue that the health care systems in most European countries are either based on a national health system or social health insurance which restricts the individual’s freedom of choice in health insurance. As a result, adverse selection does not exist in a system with compulsory insurance. However, as is also apparent from our results, there is enough variation in perceived health care cost coverage among individuals in the countries in our study. Moreover, in many of those countries, there are also private or supplementary voluntary health insurance arrangements, in parallel or in addition to national health systems or social health insurance.

Accordingly, we expect to see more effect of smoking on perceived health care cost coverage in countries where there is more choice of health care cost coverage. For instance, in countries where consumers are allowed to choose from multiple insurers or where there is the larger proportion of population who have some kinds of private health insurance. However, our results show that the negative effect of smoking on health coverage is more evident in countries where there are fewer possibilities of choice. For instance, in case of Italy or Czech Republic we find a considerable negative effect of smoking on perceived health care cost coverage. In contrast, in Germany where there is more choice both for social health insurance and private health insurance, we do not see an effect of daily smoking on perceived health care cost coverage. As noted earlier, previous studies have tried to explain the counter-conventional relationship between smoking and health insurance by heterogeneity in risk aversion. However, our results show that the negative effect of smoking on health insurance coverage cannot fully be explained by risk aversion. Because, we see this effect also in countries where there are fewer possibilities to choose health care cost coverage. One plausible explanation instead of risk aversion can be more out-of-pocket expenditures among smokers in these countries. Further research should test if this is the case.

### Self-assessed health and purchasing private health insurance

Previous studies have also found a positive correlation between self-assessed health and purchasing private health insurance in Australia (Doiron et al. 2008). Those who assess themselves as healthier are more willing to purchase private health insurance. In contrast, we find a negative effect of self-assessed health on perceived health care cost coverage but only among those who live without a partner. Thus, among them, a better perceived health decreases the level of health cost coverage. Considering self-perceived health as a proxy for health risk corroborates the notion of adverse selection in the relationship between (health) risk and (health) insurance. Other studies (Doiron et al. 2008) which find, in contrast, a positive relationship between self-assessed health and purchasing private health insurance, conclude that the correlation between self-assessed health status and purchasing private insurance is affected by other factors such as risk aversion, and socioeconomic factors which dominate the conventional adverse selection effect. However, if more objective measures of health status (i.e. the number of chronic diseases) are used, the results are consistent with the adverse selection effect. For instance, they find that those who have a chronic condition are more likely to purchase insurance and less likely to be in a good self-assessed health. These results are compatible with our findings using self-assessed health. Nevertheless, relating these findings to adverse selection should be done with caution for several reasons. First, the effect is only detected among those who live without a partner. This can be explained by heterogeneity in risk aversion between those who live with and without a partner. Second, as also mentioned in previous studies (Doiron et al. 2008), self-assessed health status can be endogenous to the insurance which makes it difficult to infer causality. Regarding the specific country results, in some countries we see a deviation from the general pattern we just discussed. For instance, in the Netherlands, we find a negative effect of self-perceived health on perceived health care cost coverage only for women with a partner while in the Austria we see the same effect among men with a partner and in Italy, a positive effect is detected.

Our study differs in several respects from earlier studies. First, it finds no effect of smoking on perceived health care cost coverage. However, for a smaller part of our sample (those who live without a partner), it identifies a causal effect of smoking on health insurance coverage which corroborates the counter-conventional relationship found in the previous study but in the language of causality. It should be noted that the identification of causality is based on our IV model which has its limitations. It cannot be considered as strong as causality inferred from an experimental design. Second, it allows for a broader sense of health cost coverage to be studied rather than only health insurance coverage. In addition, it takes into account the variation in perceived health care cost coverage between countries and also between individuals within each country. Third, it uses a subjective measure of health care cost coverage that is what individuals perceive as health care cost coverage. In fact, this perception can express the amount of protection that individuals feel they have when facing risks. This subjective measure of health care cost coverage could be more informative about the adverse selection effect than just the probability of having health insurance, because most people intuitively include perceptions in their decision making process.

### Limitations

Although an IV strategy is a common approach to deal with the endogeneity problem and to infer causality, finding a valid instrument is challenging. Given the data at hand, all our efforts failed to find more than one valid instrument. As in most studies that use instrumental variables, we have to acknowledge that our instrument variables may have some weaknesses. Based on the existing evidence on peer effect and concordant health behaviour, we assume that smoking behaviour is affected by the health behaviour of other household members (either partner or others). At the same time, we assume that the health care cost coverage of one household member is independent of the smoking behaviour of other household members. However, one may argue that the smoking habit of the partner may influence the spouse health care cost coverage. We have tested the quality of our instrument both for relevance and validity. However, it should be noted that the extent of the causal interpretation of the results depends on the extent to which the validity of the instrument can be justified. In addition, our instrument (i.e. the other household member’s smoking behaviour) is limited to the smoking behaviour of those household members who are eligible to be included in the SHARE. We do not have information about the smoking behaviour of those household members who were not eligible because of their age. For instance, if the children are still living with parents, it seems reasonable to assume that their smoking behaviour can affect their parents’ smoking behaviour. But we do not have this information at hand. Nevertheless, the results of the first stage regression show that our instrument is highly relevant.

## Conclusion

In this study, we have used, for the first time to our knowledge, an IV strategy to identify a causal effect between smoking and perceived health care cost coverage. Using data from 12 European countries, smoking is instrumented by an instrumental variable indicating whether or not there is any other daily smoker in the household, except the smokers themselves. For health care cost coverage, a self-assessment of health coverage is used which is based on the individuals’ self-assessment of the extent of the health care cost coverage they have for different types of health services. For those who live with a partner (72% of the sample), we find no effect of smoking on perceived health care cost coverage. For those who live without a partner (a smaller part of our sample), there is a negative effect of smoking on perceived health care cost coverage. In addition, this negative effect is more evident in countries where there is less variation in perceived health care cost coverage.

Thus, our study, on the one hand, show that we find no statistically significant indication of adverse selection for most of the respondents. On the other hand, when there is a statistically significant indication, the same counter-conventional relationship between smoking and perceived health care cost coverage is found as in previous studies. It can be assumed that the negative effect of smoking on perceived health care cost coverage in those countries might be because of higher out-of-pocket expenditures that a smoker may incur. This assumption needs to be tested by further research. Given that we have only one instrument, testing the overidentifying restrictions is not possible. Although at the outset, we tried to add more instruments, all our effort failed to find more relevant and valid instruments. Further research can also apply the same strategy to different datasets, looking for more than one instrument variable to identify a causal effect between health care cost coverage and smoking.
